# Assessment of sleep quality and its association with problematic internet use among university students: a cross-sectional investigation in Bangladesh

**DOI:** 10.5935/1984-0063.20200069

**Published:** 2021

**Authors:** Zohurul Islam, Kamrul Hsan, Saiful Islam, David Gozal, Mahfuz Hossain

**Affiliations:** 1 Jahangirnagar University, Department of Public Health and Informatics - Savar - Dhaka - Bangladesh.; 2 University of Missouri School of Medicine, Department of Child Health, and the Child Health Research Institute - Columbia - Missouri - United States.

**Keywords:** Sleep Quality, Problematic Internet Use, Internet Addiction, University Students, Bangladesh

## Abstract

**Objectives:**

Problematic internet use (PIU) is a major behavioral problem that has been closely associated with poor sleep quality in many different countries, but is poorly studied in Bangladesh. This study was conducted to investigate the sleep quality and its association with PIU among university students in Bangladesh.

**Material and Methods:**

A cross-sectional study was conducted between May 2019 and December 2019 among 400 students attending four public universities in Bangladesh. The Pittsburgh sleep quality index (PSQI) was used to determine sleep quality and Young’s internet addiction test (IAT) was used to describe the degree of PIU.

**Results:**

A significant negative association emerged between good sleep quality and PIU (p<0.001). In the multivariate logistic regression, students having PIU were 0.28 folds less likely to have good sleep quality (AOR: 0.28, 95%CI=0.18-0.43, p<0.001) when compared to non-PIU students. In addition, significant associations between sleep quality and socio-demographic and lifestyle factors were identified.

**Conclusion:**

Implementation of an effective awareness program and development of education strategies are required to reduce internet addictive behaviors and improve sleep quality among Bangladeshi university students.

## INTRODUCTION

It is quite obvious that nowadays, access and use of the internet have become an essential element of everyday life^1^, being widely used for entertainment, communication, and education. Globally, internet use has grown exponentially to more than 2.5 billion active users with a majority being adolescents and younger people^2^. Despite the inherent advantages afforded by internet use, excessive and uncontrolled use has become a significant social and behavioral problem. Such problematic internet use (PIU) has been termed “internet addiction” (IA) and has been traditionally defined as excessive or poorly controlled preoccupations, urges or behaviors regarding computer use and internet access that lead to impairment or distress^3^ and its prevalence varies greatly depending on the population being studied^1,4,5,6,7,8,9^.

A previous study showed that students who excessively use the internet have a greater likelihood of experiencing sleep problems, and are more likely to suffer from psychological problems than those who do not^10^. Some of the consequences of IA include depression, anxiety, stress, sleep disturbances, bad temper, restlessness, as well as problems regarding social relationships and educational achievements^5,11,12,13^. A previous study found that IA and other PIU behaviors possibly impose significant infuences on the sleep-wake schedule, leading to the emergence of insomnia and other sleep disturbances^12^. A study conducted among Chinese high school students reported that the prevalence of PIU was 17.2% among students, with 40% of students suffering from sleep disturbances, and up to 51.4% of students exhibiting depressive symptoms^14^. In Bangladesh, the prevalence of PIU was 24% among university students, with 25% of students suffering from short sleep^15^.

Sleep is one of the pillars of health and is a life-sustaining function^5,16,17^. Quality and sufficient sleep is necessary for both physical and mental health^5,13,18,19^, while poor sleep quality or insufficient sleep are closely associated with unhealthy lifestyle behaviors including internet use^20^. Indeed, excessive internet usage is a leading predictor of poor sleep quality and negligence at work^21^. Furthermore, students with internet addiction in China exhibit 1.73 times higher risk of poor sleep quality compared to other students^22^.

Sleep is also essential for cognitive functions related to academic performance in higher education^23,24,25,26,27,28,29^. In a previous study, 69.7% of college students with lower grade point average (GPA) had difficulty in falling asleep^30^. In Bangladesh, the “Digital Bangladesh” movement was launched for the promotion of information technology by the current government. Therefore, the number of internet subscribers has increased during the last few years after launching the movement, with 12.7 million new users within a single year^31^. Consequently, we are likely to witness the accelerated emergence of PIU and poor sleep quality in Bangladesh in the upcoming years. A recently published study found that the prevalence of poor sleep quality among the Bangladeshi university students is 66.6%^32^, while another study reported a prevalence of 69.5% among Bangladeshi medical college students^13^. Although a few studies have been conducted to investigate PIU among university students^15,31,33,34^, there are limited studies that evaluated potential associations between PIU and sleep quality^13^. Consequently, the study aimed to investigate the sleep quality and its association with PIU among university students in Bangladesh.

## MATERIAL AND METHODS

### Study setting and population

A cross-sectional study was conducted in four public universities (Jahangirnagar University, University of Rajshahi, Bangabandhu Sheikh Mujibur Rahman Agricultural University, and Noakhali Science & Technology University) located in Bangladesh, from May 2019 to December 2019. We recruited 400 students who met the inclusion criteria including: (1) age from 18 to 26 years; (2) regular enrolled university students; (3) having access to at least a social networking site; and (4) actively using the internet. Participants were fully voluntary and uncompensated.

### Data collection tools and technique

Data were collected through anonymous survey using a self-reported Bangla questionnaire (as participants’ first language was Bangla) consisting of three sections. Section 1 comprised questions related to socio-demographic and lifestyle variables, including age, sex, marital status, place of residence during class (living with family at home or dormitory), parents’ monthly income, father’s occupation, mother’s occupation, number of siblings, urban or rural residence prior to university, physical activity (regular or irregular), brushing of teeth before sleep (yes or no). Section 2 comprised questions to assess the degree of PIU among the university students via the Pittsburgh sleep quality index (PSQI) and section 3 comprised questions to assess sleep quality using the internet addiction test (IAT). We implemented a convenient sampling approach to recruit the study participants. Initially, 430 datasets were collected after obtaining informed consent. Of these, 400 were included in the final analysis based on inclusion criteria along with the removal of incomplete or data missing surveys.

### Pittsburgh sleep quality index (PSQI)

The PSQI is a parameter-based questionnaire relying on self-reported responses, and was first developed by Buysse et al. (1989)^35^, it is one of the popular tools for the assessment of sleep quality. PSQI consists of 19 items questions, which are grouped into seven components (i.e., subjective sleep quality, sleep latency, sleep duration, habitual sleep efficiency, use of sleep medication, and daytime dysfunction) assessed features of sleep quality over the last month^35^, each weighted equally on a 0-3 scale. The seven components scores are then summed to yield a global PSQI score of sleep quality between 0 and 21. A higher score indicates poor sleep quality^35^. The present study evaluated sleep quality among the participants by using the validated Bangla version of the PSQI, which is also called the Bengali Pittsburgh sleep quality index (BPSQI)^36^. A BPSQI score >5 was selected as the cut-off for the poor sleep quality^36,37^.

### Internet addiction test (IAT)

The IAT is a psychometric screening tool for assessing problematic internet use, developed by Young (1998)^38^ which is the first validated and reliable measure of the addictive use of internet^5,34^ measuring psychological dependence, compulsive use, and withdrawal, as well as related problems of school, sleep, family, and time management^4^. In the present study, we utilized the validated Bangla version of IAT^34^, which consists of 18 questions (e.g., *How often do you fnd that you stay online longer than you intended?*) with a five-point Likert scale ranging from 1 (rarely) to 5 (always). The validated Bangla version of IAT^34^ showed very good internal consistency (Cronbach’s α=.89), and strong convergent and discriminant validity. The total score is obtained by summating the raw score of each construct ranging from 18 to 90^34^, with the higher the score indicating the greater the level of problematic internet use. The level of internet use was categorized into three groups as minimal, moderate, and excessive users based the 18-item Bangla IAT scoring 18-35, 36-62, and 63-90, respectively^34^. In the present study, those scoring moderate to excessive scores (≥36) were classified as PIU^34^.

### Statistical analysis

Data were analyzed using the Statistical Package for Social Science (SPSS) software version 25.0. Statistical analyses included descriptive statistics (e.g., frequencies, percentages, means, Chi-square test and Fisher’s exact test). Categorical variables were compared between two groups “good sleep quality” vs. “poor sleep quality”. Logistic regression (both unadjusted and adjusted models) was performed with 95% confidence intervals to determine significant associations between categorical dependent and independent variables. Analyses were univariate, crude odds ratios (COR) of all examined variables (independent) were compared with sleep quality as the dependent variable. Multivariable analyses were also performed with all variables combined in the model as covariates to yield adjusted odds ratios (AOR) where the dependent variable included sleep quality. A *p-*value of less than 0.05 was considered as statistically significant.

### Ethical considerations

This study was approved by the Biosafety, Biosecurity and Ethical Committee of the Jahangirnagar University, Savar, Dhaka-1342, Bangladesh [Ref. No.: BBEC, JU/M2020 (2)2]. Informed written consent was obtained from all the participants prior to data collection. The objectives of the research were explained to the participants and they were informed that they could choose to participate (or not) in the study. Only after getting their permission to participate in the study, they were surveyed. Strict confidentiality of information and anonymity to the participants was ensured.

## RESULTS

400 university students aged 18-26 years of which 279 (69.8%) were male completed the survey. The average age of participants was 21.2 years (SD=1.8) and most of the participants came from rural areas (61.5%). Most of the participants were single (94.3%) and stayed in the university student dormitory (64.3%); 86% were non-smokers, 80.3% did not perform physical exercise regularly, and 51.2% were not brushing teeth before sleep. Of the respondents, 67.3% had access to internet through mobile, 11% through laptop, and 21.8% through both devices. Based on IAT scores, the prevalence rates of minimal, moderate, and excessive users were 46.0%, 47.8%, and 6.3%, respectively, that indicating 54% participants fulfilled the criteria as PIU. In addition, 51.5% of study participants reported good sleep quality (Table 1).

**Table 1:** Socio-demographic and lifestyle factors among 400 university students.

Variables	Categories	Frequency	Percentage (%)
**Age**	18-20	167	(41.8)
	21-23	181	(45.3)
	24-26	52	(13.0)
**Sex**	Male	279	(69.8)
	Female	121	(30.3)
**Marital status**	Married	23	(5.8)
	Single	377	(94.3)
**Place of residence**	Home	143	(35.8)
	Hall	257	(64.3)
**Father's occupation**	Services	173	(43.3)
	Businessman	95	(23.8)
	Farmer	99	(24.8)
	Immigrant	6	(1.5)
	Dead	27	(6.8)
**Mother's occupation**	Housewife	328	(82.0)
	Employed	61	(18.0)
**Monthly family income**	<50000 BDT	340	(85.0)
	>50000 BDT	60	(15.0)
**No of siblings**	1-3	303	(75.8)
	4-6	90	(22.5)
	>7	7	(1.8)
**Came from**	Rural area	246	(61.5)
	Urban area	154	(38.5)
**Internet browsing device**	Mobile	269	(67.3)
	Laptop	44	(11.0)
	Both devices	87	(21.8)
**Smoking habits**	Yes	56	(14.0)
	No	344	(86.0)
**Physical activity**	Regular	79	(19.8)
	Irregular	321	(80.3)
**Brushing teeth before sleep**	Yes	195	(48.8)
	No	205	(51.2)
**Internet use**	Non-PIU	184	(46.0)
	PIU	216	(54.0)
**Sleep quality**	Good	206	(51.5)
	Poor	194	(48.5)

Note: BDT = Bangladeshi Taka.

There were no significant differences between sleep quality and age groups, gender, marriage status, residence location and origin, physical activity, smoking habits, or other demographic factors. However, participants’ sleep quality was associated with their mothers’ occupation (χ^2^=6.67, df=1, *p*=0.01) and internet use (χ^2^=36.85, df=1, *p*<0.001) (Table 2).

**Table 2:** Associations between socio-demographic and lifestyle factors, internet use, and sleep quality among university students (N=400).

Variables	Categories	Sleep quality				*χ^2^*	*df*	*p-*value
		Poor		Good				
		n	(%)	n	(%)			
**Age (years)**	18-20	80	(41.2)	87	(42.2)	0.247	2	0.884
	21-23	90	(46.4)	91	(44.2)			
	24-26	24	(12.4)	28	(13.6)			
**Sex**	Male	143	(73.7)	136	(66.0)	2.802	1	0.094
	Female	51	(26.3)	70	(34.0)			
**Marital status**	Married	13	(6.7)	10	(4.9)	0.629	1	0.428
	Single	181	(93.3)	196	(95.1)			
**Place of residence**	With family at home	78	(40.2)	65	(31.6)	3.257	1	0.071
	Dormitory	116	(59.8)	141	(68.4)			
**Father's occupation**	Services	78	(40.2)	95	(46.1)	2.216†	4	0.706
	Businessman	49	(25.3)	46	(22.3)			
	Farmer	51	(26.3)	48	(23.3)			
	Immigrant	2	(1.0)	4	(1.9)			
	Dead	14	(7.2)	13	(6.3)			
**Mother's occupation**	Housewife	169	(87.1)	159	(77.2)	6.673	1	**0.010**
	Employed	25	(12.9)	47	(22.8)			
**Monthly family income**	<50000 BDT	169	(87.1)	171	(83.0)	1.320	1	0.251
	>50000 BDT	25	(12.9)	35	(17.0)			
**No of siblings**	1-3	148	(76.3)	155	(75.2)	0.182†	2	0.973
	4-6	43	(22.2)	47	(22.8)			
	>7	3	(1.5)	4	(1.9)			
**Came from**	Rural area	120	(61.9)	126	(61.2)	0.020	1	0.887
	Urban area	74	(38.1)	80	(38.8)			
**Internet browsing device**	Mobile	127	(65.5)	142	(68.9)	3.325	2	0.190
	Laptop	18	(9.3)	26	(12.6)			
	Both devices	49	(25.3)	38	(18.4)			
**Smoking habits**	Yes	29	(14.9)	27	(13.1)	0.281	1	0.596
	No	165	(85.1)	179	(86.9)			
**Physical activity**	Regular	37	(19.1)	42	(20.4)	0.109	1	0.741
	Irregular	157	(80.9)	164	(79.6)			
**Brushing teeth before sleep**	Yes	97	(50.0)	98	(47.6)	0.236	1	0.627
	No	97	(50.0)	108	(52.4)			
**Internet use**	Non-PIU	59	(30.4)	125	(60.7)	36.847	1	**<0.001**
	PIU	135	(69.6)	81	(39.3)			

Bivariate and multivariate logistic regression analyses were performed and the unadjusted and adjusted odds ratios are shown in Table 3. In the unadjusted model, students having PIU were 0.28 times less likely to have good sleep quality than non-PIU (AOR: 0.28, 95%CI=0.19-0.43, *p*<0.001). In addition, the adjusted model indicated that PIU was negatively associated with good sleep quality. Students reporting PIU were 28% less likely to have good sleep quality (AOR: 0.28; 95%CI=0.18-0.43, *p*<0.001) when compared to non-PIU. The model also indicated that the respondents whose mother was a housewife were 50% less likely to have good sleep quality than respondents whose mother was employed (COR: 0.50, 95%CI=0.29-0.85, *p*=0.011) but this was insignificant in adjusted model (Table 3).

**Table 3:** Bivariate and multivariate analysis of factors associated with sleep quality.

Variables	Categories	Unadjusted model^a^				Adjusted model^b^	
		COR	95%CI	*p-*value AOR		95%CI	*p-*value
**Age**	18-20	0.93	(0.49-1.74)	0.825	1.09	(0.52-2.29)	0.827
	21-23	0.87	(0.47-1.61)	0.650	0.80	(0.39-1.62)	0.528
	24-26	Reference			Reference		
**Sex**	Male	0.69	(0.45-1.07)	0.095	0.81	(0.49-1.32)	0.393
	Female	Reference			Reference		
**Marital status**	Married	1.41	(0.60-3.29)	0.430	0.74	(0.29-1.87)	0.520
	Single	Reference			Reference		
**Place of residence**	Home	0.69	(0.46-1.03)	0.072	0.68	(0.43-1.10)	0.114
	Student hall	Reference			Reference		
**Father's occupation**	Services	1.31	(0.58-3.00)	0.513	1.28	(0.53-3.07)	0.585
	Businessman	1.01	(0.43-2.38)	0.980	1.20	(0.47-3.04)	0.700
	Farmer	1.01	(0.43-2.38)	0.975	0.98	(0.38-2.54)	0.962
	Immigrant	2.15	(0.34-13.80)	0.418	1.67	(0.23-11.82)	0.610
	Dead	Reference			Reference		
**Mother's occupation**	Housewife	0.50	(0.29-0.85)	0.011	0.54	(0.30-1.00)	0.050
	Employed	Reference			Reference		
**Monthly family income**	<50000 BDT	0.72	(0.42-1.26)	0.25	0.56	(0.29-1.08)	0.085
	>50000 BDT	Reference			Reference		
**No of siblings**	1-3	0.79	(0.17-3.57)	0.755	0.48	(0.10-2.42)	0.374
	4-6	0.82	(0.17-3.87)	0.802	0.55	(0.10-2.86)	0.474
	>7	Reference			Reference		
**Came from**	Rural area	0.97	(0.65-1.45)	0.887	1.17	(0.70-1.92)	0.576
	Urban area	Reference			Reference		
**Internet browsing device**	Mobile	1.44	(0.89-2.35)	0.141	1.63	(0.94-2.82)	0.082
	Laptop	1.86	(0.89-3.89)	0.097	1.50	(0.67-3.39)	0.325
	Both devices	Reference			Reference		
**Smoking habit**	Yes	0.86	(0.49-1.51)	0.596	0.83	(0.43-1.70)	0.583
	No	Reference			Reference		
**Physical exercise**	Regular	1.09	(0.66-1.78)	0.741	1.11	(0.63-1.94)	0.724
	Irregular	Reference			Reference		
**Brushing teeth before sleep**	Yes	0.91	(0.61-1.34)	0.627	0.87	(0.56-1.36)	0.546
	No	Reference			Reference		
**Internet use**	PIU	0.28	(0.19-0.43)	<0.001	0.28	(0.18-0.43)	<0.001
	Non-PIU	Reference			Reference		

Notes: BDT = Bangladeshi Taka; COR = Crude Odds Ratio; CI = Confidence Interval; AOR = Adjusted Odds Ratio.

a Unadjusted Model: Dependent variable: Sleep quality; Independent variables: Age, Sex, Marital status, Place of residence, Father's occupation, Mother's occupation, Monthly family income, No of siblings, Came from, Internet browsing device, Smoking Habit, Physical exercise, Brushing teeth before sleep, and Internet use;

b Adjusted Model: Dependent variable: Sleep quality; Covariates: Age, Sex, Marital status, Place of residence, Father's occupation, Mother's occupation, Monthly family income, No of siblings, Came from, Internet browsing device, Smoking Habit, Physical exercise, Brushing teeth before sleep, and Internet use.

## DISCUSSION

The present study showed that the presence of PIU and poor sleep quality is highly prevalent and correlated among university students in Bangladesh. Evidence suggests that PIU is significantly associated with depression, poor sleep quality, mood changes, and unfavorable health outcomes, such as obesity and low self-esteem^34,39^. Students who use internet excessively have an increased chance of experiencing sleep problems^26^. Therefore, sleep disturbances among university students are not only more likely to occur but carry also a potentially significant burden among students who spend excessive hours surfing the internet^5^. Although a few studies have already investigated the prevalence of PIU among university students in Bangladesh^15,31,33,34^, studies assessing the association between PIU and sleep quality are very limited^13^.

The prevalence of poor sleep quality in the present study was comparatively lower than in the only other study in Bangladesh, which reported poor sleep quality in 69.5% of medical students^13^. Similarly, Afandi et al. (2017)^40^ reported poor sleep quality in 67.2% of university students in the United Arab Emirates (UAE). The reasons for such discrepant findings are unclear but could refect both the type of students (medical vs. others) or the differences in overall ease of access to the internet in different countries. The majority of the respondents in the present study were moderate internet users (36.8%), with 6.3% being considered excessive internet users. Differences in the frequency of PIU among university students have been reported in different studies in Bangladesh, whereby Karim and Nigar (2014)^34^ found only 1.7% as being excessive users, while and Islam and Hossin (2016)^15^, found that 24% were problematic users (i.e., either moderate or excessive internet users) and Mamun et al. (2019)^31^ found that 3.9% of graduate students were excessive internet users^15,31,34^. Thus, the escalation of the number of users over the years and the overall duration of use could have contributed to the differences across the various studies.

The proportion of poor sleep quality among male university students was higher than among female students, although there is no significant gender difference regarding overall sleep quality. Other studies conducted in Turkey^41^ and Bangladesh^13^ also found a higher prevalence of poor sleep quality among male students. Students whose mothers were homemakers and were not in paid employment were less likely to have good sleep quality than employed mothers, which supports the prior findings^13^. Elucidation of this particular issue will have to await additional studies.

In this study, we found a significant association between PIU and sleep quality among university students, suggesting that increasing or excessive internet use may favor the emergence of poor sleep quality. Of note, a prior Bangladeshi study conducted among medical college students reported a significant negative association between PIU and good sleep quality^13^. However, the findings of this study were subsequently criticized due to methodological limitations that involved the fact that the English language instrument (i.e., the Orman’s Internet Addiction Survey): (i) was never published in a peer-reviewed journal; (ii) never underwent any psychometric testing (not even basic reliability or validity checks); (iii) has cut-off scores that have never been subjected to specificity or sensitivity analyses; and (iv) was developed over two decades ago and is therefore outdated^42^. The reason for this finding could be that increased internet use leads to virtual connectivity with more individuals via Facebook, WeChat, WhatsApp Twitter, LinkedIn, and other online social media, which in turn enhances further reliance on the internet, with such time on the internet displacing sleep.

In addition, increased internet use could increase spending time reading online news and watching videos on YouTube, also displacing sleep. Our findings are concurrent with other studies around the world that also found significant associations between PIU and sleep quality among students^2,5,13,20,22,43,44^. A study in Taiwanese college students also reported that poor sleep quality was 1.4 folds more likely to be experienced by those students with PIU^45^. Similarly, it has been reported that most students with sleep problems will be spending more time online and watching television^46^.

The study will help to explore the research gap more rigorously in different settings, and results from such studies should infor m new policies regarding how Bangladeshi university students’ sleep characteristics are being affected by problematic internet use. Current findings suggest that an immediate intervention to this vulnerable group aimed at improving sleep quality by reducing problematic internet use should be beneficial. However, systematic online counseling, education leading to increased awareness, and methodologies aimed at improving motivation need to be developed in this respect.

### Strengths and limitations

This study is one of the few reporting on the association between sleep and PIU, and used previously validated tools to this effect. However, the study is limited by the use of self-reported information regarding internet use or sleep problems which might have infuenced the results through well-known biases (i.e., memory recall biases and social desirability biases). Furthermore, the study was cross-sectional in nature, and therefore cannot provide any indication of causality. The study was also limited by relatively a small sample size, and it was conducted at only four Universities in Bangladesh; therefore, generalizability to other university samples (and other types of student populations) in the country may be limited. Future studies should overcome such limitations by employing longitudinal designs with larger and more representative samples.

## CONCLUSION

PIU and reduced sleep quality among university students in Bangladesh are likely to co-exist and potentially lead to adverse consequences regarding health and academic performance. Implementation of effective awareness programs and the development of education strategies for university students appear to be highly advisable to attenuate the impact of internet addiction and improve sleep quality among university students.
